# Circulating microRNA Panel for Prediction of Recurrence and Survival in Early-Stage Lung Adenocarcinoma

**DOI:** 10.3390/ijms25042331

**Published:** 2024-02-16

**Authors:** Mei-Chee Tai, Leonidas E. Bantis, Gargy Parhy, Taketo Kato, Ichidai Tanaka, Chi-Wan Chow, Junya Fujimoto, Carmen Behrens, Tetsunari Hase, Koji Kawaguchi, Johannes F. Fahrmann, Edwin J. Ostrin, Kohei Yokoi, Toyofumi F. Chen-Yoshikawa, Yoshinori Hasegawa, Samir M. Hanash, Ignacio I. Wistuba, Ayumu Taguchi

**Affiliations:** 1 Department of Translational Molecular Pathology, The University of Texas MD Anderson Cancer Center, Houston, TX 77030, USAgargy.parhy@gmail.com (G.P.); taketokato63@gmail.com (T.K.);; 2 Department of Biostatistics and Data Science, University of Kansas Medical Center, Kansas City, KS 66160, USA; 3 Department of Thoracic Surgery, Nagoya University Graduate School of Medicine, Nagoya 466-8560, Japan; k-gucci@clin.medic.mie-u.ac.jp (K.K.); tyoshikawa@med.nagoya-u.ac.jp (T.F.C.-Y.); 4 Department of Respiratory Medicine, Nagoya University Graduate School of Medicine, Nagoya 466-8560, Japanthase@med.nagoya-u.ac.jp (T.H.); yhasega@med.nagoya-u.ac.jp (Y.H.); 5 Department of Thoracic/Head and Neck Medical Oncology, The University of Texas MD Anderson Cancer Center, Houston, TX 77030, USA; 6 Department of Clinical Cancer Prevention, The University of Texas MD Anderson Cancer Center, Houston, TX 77030, USA; jffahrmann@mdanderson.org (J.F.F.); shanash@mdanderson.org (S.M.H.); 7 Department of Pulmonary Medicine, The University of Texas MD Anderson Cancer Center, Houston, TX 77030, USA; 8 National Hospital Organization Nagoya Medical Center, Nagoya 460-0001, Japan; 9 Division of Molecular Diagnostics, Aichi Cancer Center, Nagoya 464-8681, Japan; 10 Division of Advanced Cancer Diagnostics, Nagoya University Graduate School of Medicine, Nagoya 466-8560, Aichi, Japan

**Keywords:** lung adenocarcinoma, plasma, microRNA, recurrence, survival

## Abstract

Early-stage lung adenocarcinoma (LUAD) patients remain at substantial risk for recurrence and disease-related death, highlighting the unmet need of biomarkers for the assessment and identification of those in an early stage who would likely benefit from adjuvant chemotherapy. To identify circulating miRNAs useful for predicting recurrence in early-stage LUAD, we performed miRNA microarray analysis with pools of pretreatment plasma samples from patients with stage I LUAD who developed recurrence or remained recurrence-free during the follow-up period. Subsequent validation in 85 patients with stage I LUAD resulted in the development of a circulating miRNA panel comprising miR-23a-3p, miR-320c, and miR-125b-5p and yielding an area under the curve (AUC) of 0.776 in predicting recurrence. Furthermore, the three-miRNA panel yielded an AUC of 0.804, with a sensitivity of 45.8% at 95% specificity in the independent test set of 57 stage I and II LUAD patients. The miRNA panel score was a significant and independent factor for predicting disease-free survival (*p* < 0.001, hazard ratio [HR] = 1.64, 95% confidence interval [CI] = 1.51–4.22) and overall survival (*p* = 0.001, HR = 1.51, 95% CI = 1.17–1.94). This circulating miRNA panel is a useful noninvasive tool to stratify early-stage LUAD patients and determine an appropriate treatment plan with maximal efficacy.

## 1. Introduction

One-third of patients with non-small cell lung cancer (NSCLC) are diagnosed at an early stage and have the potential to undergo a curative-intent surgical resection procedure [1]. However, that report revealed that five-year survival rates ranged from 83% for stage IA (The American Joint Committee on Cancer [AJCC] 7th Edition) to 49% for stage IIB (AJCC 7th Edition). Therefore, patients with early-stage NSCLC still face a 20–50% risk of recurrence or disease-related death, despite recent improvements in early detection and therapeutic strategies. Cisplatin-based adjuvant chemotherapy remains the standard care for patients who have undergone NSCLC resection with a substantial risk of recurrence [2,3,4]. A large meta-analysis of randomized clinical trials compared adjuvant chemotherapy with patients who received a placebo or only received clinical observations, and found that cisplatin-based adjuvant chemotherapy led to a significant improvement in 5-year overall survival (OS) of 5% [3]. However, adjuvant chemotherapy has been associated with an increased risk of substantial toxicity, including chemotherapy-related death, and provides only modest survival benefits [5,6]. Therefore, there is a critical unmet need for biomarkers to assess the risk of recurrence in early-stage NSCLC patients and identify those who would be more likely to benefit from adjuvant chemotherapy.

We and others have presented molecular profiles in tumor tissues as promising prognostic biomarkers [7,8,9]. However, because of substantial intra-tumor heterogeneity [10], information from a single biopsy sample may not accurately reflect the biological characteristics of a tumor. As a result, the assessment of blood-based biomarkers, such as circulating tumor DNAs (ctDNAs), proteins, and autoantibodies, has emerged as a promising approach for minimally invasive recurrence prediction in NSCLC that may overcome the issues of sampling bias associated with single-tumor biopsy analysis [11,12,13]. However, none of these molecular biomarkers have yet been translated into clinical practice. MicroRNAs (miRNAs) are involved in multiple biological functions through post-transcriptional gene silencing and have been found to be highly deregulated in various types of cancer, including NSCLC [14]. Circulating miRNAs are stably present in blood and potentially reflect different expressions in cancerous and non-cancerous tissues, making them attractive biomarkers [15]. To date, several circulating miRNAs, such as miR-21-5p and miR-125b-5p, have been shown to be associated with the outcome of NSCLC patients [15,16,17].

In the present study, we performed miRNA microarray profiling of plasma collected before surgery from patients with stage I lung adenocarcinoma (LUAD) who developed recurrence within two years after curative surgery or remained recurrence-free over a six-year follow-up period. LUAD is the most common histological subtype of NSCLC, accounting for more than 50% of all NSCLC cases [18]. Based on assessments of the predictive potential of miRNA candidates selected from miRNA microarray data using independent plasma sample sets and from previous reports, a circulating miRNA panel was developed and independently validated for risk prediction of recurrence and poor overall survival in patients with early-stage LUAD.

## 2. Results

### 2.1. Comparison of Plasma miRNA Profiles of Stage I LUAD Patients with or without Recurrence

**Figure 1:**
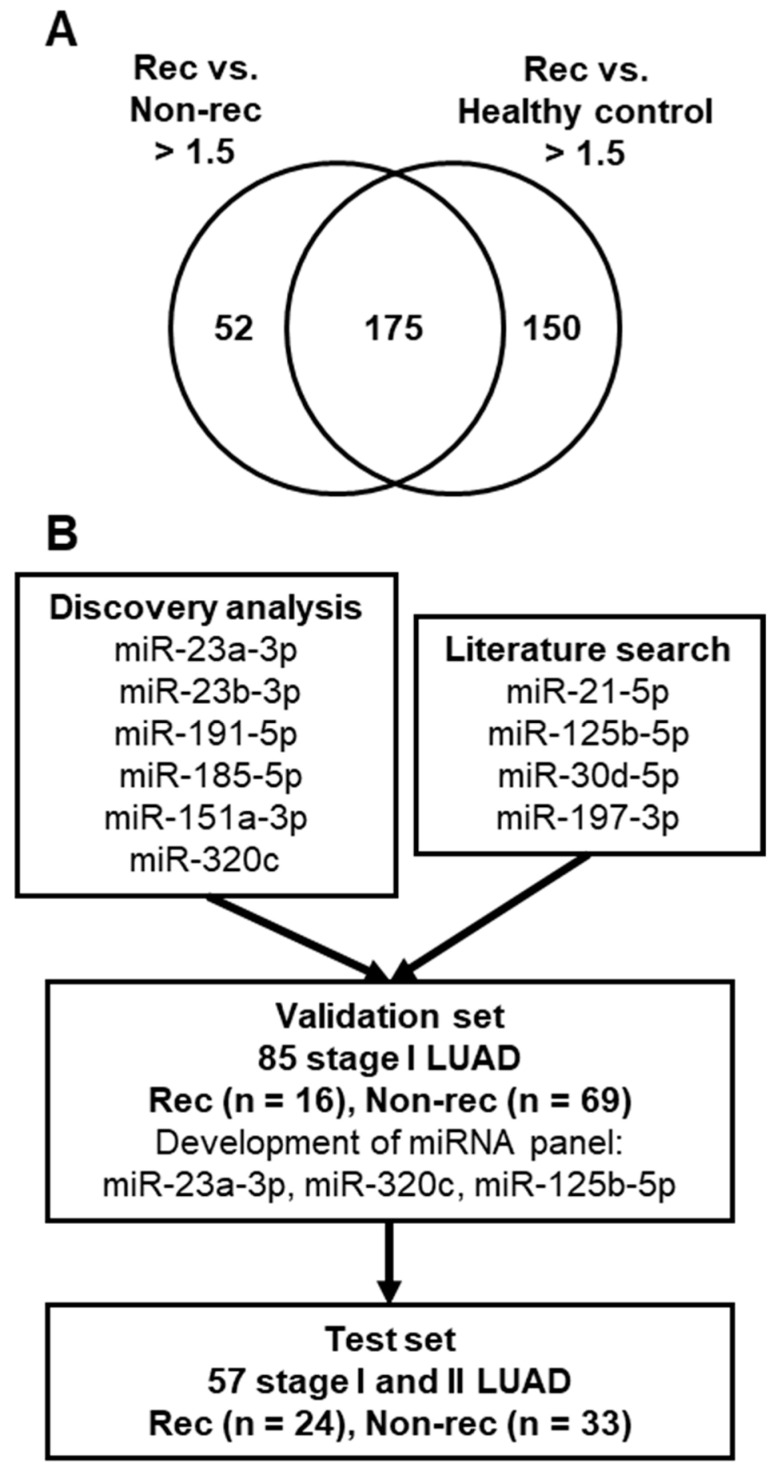
Identification of circulating miRNAs for recurrence prediction in stage I LUAD patients. (**A**) Overlap of miRNAs with 1.5-fold increase in comparison between the rec and non-rec groups and between the rec group and healthy controls. (**B**) Schematic diagram showing study design.

To identify circulating miRNAs associated with recurrence, we performed miRNA microarray analyses of pools of plasma obtained from stage I LUAD patients who either subsequently developed recurrence within two years after curative surgery (rec group) or remained recurrence-free over a six-year follow-up period (non-rec group), as well as from healthy controls. Plasma miRNA profiles were compared between the rec and non-rec groups and between the rec group and healthy controls. A total of 175 miRNAs that exhibited a greater than 1.5-fold increase were identified in the rec group compared to the non-rec group and healthy controls (Figure 1A). To identify miRNAs potentially derived from tumor cells, 19 miRNAs highly expressed in blood cells were excluded [19]. Furthermore, the expression levels of miRNAs in publicly available microarray datasets of H1299 LUAD cell lines [20] and peripheral blood mononuclear cells (PBMCs) [21] were assessed. The expression levels of 15 of 156 miRNAs were greater than 1.5-fold higher in H1299 LUAD cells compared to the PBMCs (Table 1). Based on additional evaluation of candidate miRNA expression levels demonstrated in our previous qRT-PCR array results of 34 LUAD cell lines [22] as well as the qRT-PCR results of 10 LUAD cell lines (Table 1), miR-23a-3p, miR-23b-3p, miR-191-5p, miR-185-5p, miR-151a-3p, and miR-320c were selected for further testing. In addition, the potential of additional miRNAs with supportive evidence demonstrating their potential as prognostic biomarkers for NSCLC was determined so as to yield an miRNA panel useful for the prediction of recurrence in LUAD cases. Based on prior reports [15,16,17] and expression levels in those 34 LUAD cell lines [22], miR-21-5p, miR-125b-5p, miR-30d-5p, and miR-197-3p were included for testing in the validation set (Figure 1B).

### 2.2. Validation of miRNA Biomarker Candidates and Development of Circulating miRNA Panel Using Plasma Samples from Stage I LUAD Patients

**Figure 2:**
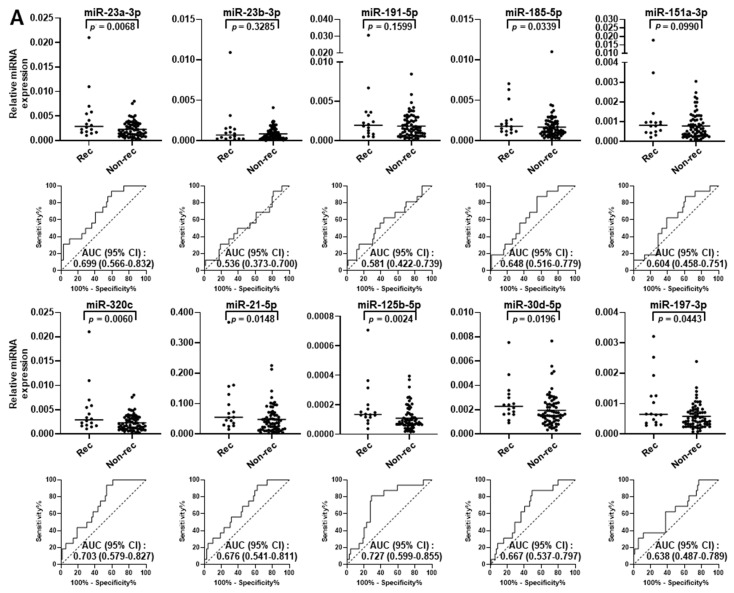

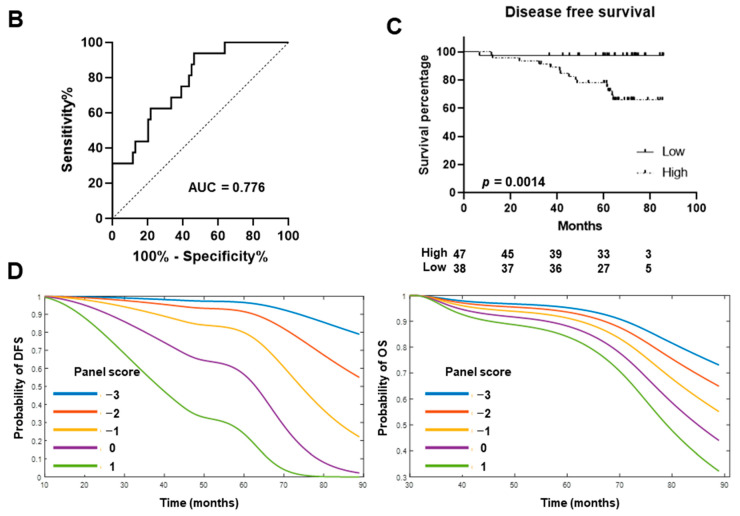
Performance of circulating miRNAs in validation set. (**A**) Circulating levels and receiver operating characteristic (ROC) curves of miR-23a-3p, miR-23b-3p, miR-191-5p, miR-185-5p, miR-151a-3p, miR-320c, miR-21-5p, miR-125b-5p, miR-30d-5p, and miR-197-3p in plasma samples collected before curative surgery from stage I LUAD patients who had recurrence (rec, *n* = 16) or who remained recurrence-free (non-rec, *n* = 69) during the six year follow-up period. Horizontal lines indicate the median. The dashed diagonal line indicates the null hypothesis (AUC = 0.500). *p* values were calculated using Mann–Whitney U test. AUC: area under the curve. (**B**) ROC curves for combined panel of miR-23a-3p, miR-320c, and miR-125b-5p in validation set. (**C**) Kaplan–Meier survival curves for disease-free survival in validation set. LUAD patients were dichotomized according to the cutoff values based on the Youden index (high, *n* = 47; low, *n* = 38). *p* values were calculated using a log-rank test. (**D**) Estimation of probability of DFS and OS based on miRNA panel scores. DFS: disease-free survival. OS: overall survival.

The individual predictive performance of circulating miR-23a-3p, miR-23b-3p, miR-191-5p, miR-185-5p, miR-151a-3p, miR-320c, miR-21-5p, miR-125b-5p, miR-30d-5p, and miR-197-3p was evaluated in an independent set of plasma samples collected prior to surgery from 85 patients with stage I LUAD (validation set; Table 2). Of those patients, 16 (18.8%) had recurrence during the follow-up period. Clinical factors as well as CEA levels were not significantly different between LUAD patients with and without recurrence (Table 2). Only two patients in the validation set, one with recurrence and one without, received adjuvant chemotherapy. In contrast, the plasma levels of miR-23a-3p, miR-185-5p, miR-320c, miR-21-5p, miR-125b-5p, miR-30d-5p, and miR-197-3p were significantly higher in those with recurrence (*p* = 0.0068, *p* = 0.0339, *p* = 0.0285, *p* = 0.0060, *p* = 0.0148, *p* = 0.0024, *p* = 0.0196, respectively, Mann–Whitney U test) (Figure 2A). Each of these seven miRNAs yielded an area under the curve (AUC) greater than 0.600 (0.699, 0.648, 0.703, 0.676, 0.727, 0.667, 0.638, respectively).

Next, we sought to develop amiRNA panel for prediction of recurrence in stage I LUAD patients based on a logistic regression model. The combination of miR-23a-3p, miR-320c, and miR-125b-5p yielded an AUC of 0.776 (95% confidence interval [CI] = 0.660 to 0.893) (Figure 2B). The LUAD patients were divided into high- (*n* = 47) and low-risk (*n* = 38) groups according to the Youden index-based optimal cutoff point, with a corresponding sensitivity of 93.8% and specificity of 53.6%. Kaplan–Meier survival curves revealed that stage I LUAD patients with high risk had significantly worse disease-free survival (DFS) (Figure 2C; *p* = 0.0014, log-rank test). As shown in Figure 2D, miRNA panel scores could distinctively stratify stage I LUAD patients based on risk of recurrence (left panel) or death (right panel), suggesting the potential of this miRNA panel to predict outcomes for LUAD cases.

### 2.3. Testing of miRNA Panel in an Independent Set of Early-Stage LUAD Plasma Samples

**Figure 3:**
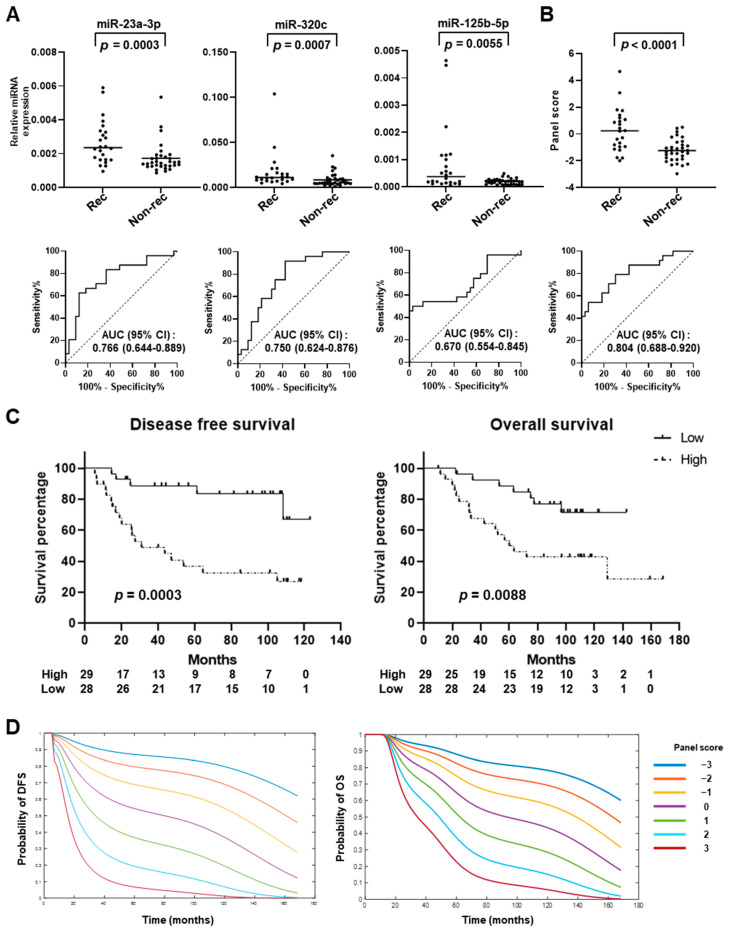
Blinded validation of miRNA panel in test set. (**A**) Circulating levels and receiver operating characteristic (ROC) curves of miR-23a-3p, miR-320c, and miR-125b-5p in plasma samples collected before curative surgery from early-stage LUAD patients who had recurrence (rec, *n* = 24) or who remained recurrence-free (non-rec, *n* = 33) during the follow-up period. The dashed diagonal line indicates the null hypothesis (AUC = 0.500). (**B**) Predictive performance of miRNA panel scores in test set. (**C**) Kaplan–Meier survival curves for disease-free and overall survival in test set. LUAD patients were dichotomized according to the cutoff values based on the Youden index (high, *n* = 29; low, *n* = 28). *p* values were calculated using a log-rank test. (**D**) Estimation of probability of DFS and OS based on miRNA panel scores. (**A**,**B**) Horizontal lines indicate median. *p* values were calculated using Mann–Whitney U test. AUC: area under the curve. DFS: disease-free survival. OS: overall survival.

Next, blinded validation of the panel of miR-23a-3p, miR-320c, and miR-125b-5p using fixed coefficients from the logistic regression model was conducted using an independent set of plasma samples obtained from 57 stage I and II LUAD patients with (*n* = 24) or without (*n* = 33) recurrence (test set) (Figure 1B, Table 2). In the test set, only the stage was significantly associated with recurrence (*p* = 0.0058, Fisher’s exact test) (Table 2 and Table S1). Notably, the levels of all three circulating miRNAs were significantly higher in plasma samples from early-stage LUAD patients with recurrence compared to those without (miR-23a-3p: *p* = 0.0003, miR-320c: *p* = 0.0007, miR-125b-5p: *p* = 0.0055, Mann–Whitney U test), yielding AUCs of 0.766, 0.750, and 0.670, respectively (Figure 3A). The miRNA panel with fixed coefficients was applied to the test set and the resulting scores were also significantly higher for early-stage LUAD patients with recurrence compared to without (*p* = 0.0009, Mann–Whitney U test); the panel yielded an AUC of 0.804 (95% CI = 0.688 to 0.920) with a sensitivity of 45.8% at 95% specificity (Figure 3B).

**Figure 4:**
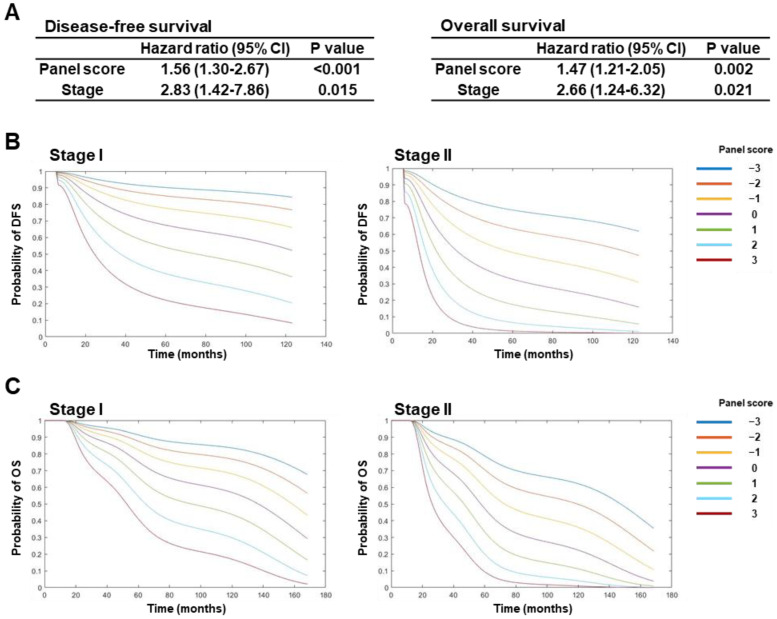
miRNA panel score by stage in test set. (**A**) Multivariate cox proportional hazard analysis of disease-free survival (left) and overall survival (right) in test set. Stage was included as a co-variable in the multivariate models. Estimation of probability of (**B**) DFS or (**C**) OS with miRNA panel score by stage. DFS: disease-free survival. OS: overall survival.

LUAD patients were divided into high- (*n* = 29) and low-risk (*n* = 28) groups according to the Youden index-based optimal cutoff point with a corresponding sensitivity of 79.2% and specificity of 69.7%. Kaplan–Meier survival curves demonstrated that higher miRNA panel scores were significantly associated with both worse DFS (*p* = 0.0003, log-rank test) and overall survival (OS) (*p* = 0.0088; log-rank test) (Figure 3C). Consistent with the findings obtained in the validation set, scores with the miRNA panel stratified early-stage LUAD patients based on risk of recurrence and death (Figure 3D). In addition, multivariable Cox regression analyses with stage included as a co-variable demonstrated that the miRNA panel score was a significant and independent predictor of DFS (*p* < 0.001, HR = 1.56, 95% CI = 1.30–2.67) and OS (*p* = 0.0002, HR = 1.47, 95% CI = 1.21–2.05) (Figure 4A). The results of the DFS and OS trajectories based on stage, as well as the miRNA panel scores, are presented in Figure 4B,C.

## 3. Discussion

In this study, circulating miRNAs in pretreatment plasma samples from stage I LUAD patients who subsequently developed recurrence after curative surgery or remained recurrence-free for up to six years were profiled using a microarray to identify potential miRNA biomarkers for predicting recurrence in early-stage LUAD. Based on the integration of miRNA expression profiles in LUAD cell lines and results of a literature search, 10 miRNAs were selected and assayed via qRT-PCR using plasma samples from 85 stage I LUAD patients, resulting in the validation of seven of them and the subsequent development of a circulating miRNA panel comprising miR-23a-3p, miR-320c, and miR-125b-5p. This three-miRNA panel was further validated in an independent plasma sample set from 59 stage I and II LUAD patients. While high levels of circulating miR-125b-5p have been associated with poor outcomes in NSCLC [15], the present results are the first to show that levels of miR-23a-3p and miR-320c in two independent plasma sample sets were significantly increased in early-stage LUAD patients who developed recurrence after curative surgery compared to those who did not.

miR-23a-3p is a constituent of the miR-23a/24/27a cluster and has been shown to be dysregulated in different types of cancer, including liver [23], colorectal [24], acute myelogenous leukemia [25], and lung cancer [26]. miR-23a-3p has an oncogenic role in these types of cancer, with direct targeting of PGC-1α and G6PC [23], MTSS1 [24], and RKIP [25], as well as suppressor genes of both the Wnt/β-catenin signaling pathway and DNA methylation pathway in cooperation with other miRNAs in the miR-23a/24/27a cluster [26]. Emerging evidence has also suggested the potential of circulating miR-23a-3p either alone or in combination with other circulating miRNAs as a diagnostic or prognostic biomarker in cancer patients [27]. The levels of miR-23a-3p in circulating EpCAM^+^ extracellular vesicles (EVs) were increased in colorectal cancer patients compared to healthy controls and they then decreased after surgery [28]. Furthermore, a 4-miRNA ratio model including miR-23a-3p was found to be significantly associated with postoperative biochemical recurrence in prostate cancer patients [29]. Of note, Fan et al. recently demonstrated that increased expression levels of the miR-23a/24/27a cluster in early-stage NSCLC tumor tissues were significantly associated with recurrence and poor prognosis [26], which is in line with the present findings showing a significant association of increased miR-23a-3p levels in plasma with recurrence in early-stage LUAD patients who underwent curative surgery.

While the expression pattern and function of miR-320c in cancer have not been well studied, Peng et al. reported that levels of EV-derived miR-320c were significantly increased in pretreatment plasma from EGFR/ALK-negative NSCLC patients who were resistant to anti-PD-1 immunotherapy compared with those who demonstrated a response [30]. Other reports have noted that miR-320a-3p, an miR-320 family miRNA, promoted the polarization of macrophages to the immunosuppressive M2 phenotype, while lung cancer patients with higher levels of EV-derived miR-320a-3p exhibited shorter overall survival [31,32]. Given that miR-320a-3p shares 19 ribonucleotide RNA sequences with miR-320c, miR-320a-3p and miR-320c likely regulate similar target RNAs and miR-320c may have an immunosuppressive role in LUAD. Interestingly, the other two miRNAs in the present panel also possess immunosuppressive functions. miR-23a-3p, induced by tumor-derived TGF-β, has been reported to suppress the cytotoxicity of tumor-infiltrating CD8^+^ cytotoxic T lymphocytes through the direct repression of BLIMP-1 [33], and miR-125b-5p has been found to suppress γδ T-cell cytotoxicity against tumor cells [34]. These findings suggest a functional relevance of immunosuppressive miRNAs in LUAD development and the potential of circulating miRNAs to predict clinical outcomes, including response to immune checkpoint inhibitors, as recently indicated [35].

The logistic regression model, comprising circulating miR-23a-3p, miR-320c, and miR-125b-5p, yielded an AUC of 0.804 with a sensitivity of 45.8% at 95% specificity for discriminating between early-stage LUAD patients who developed recurrence after curative surgery and those who remained recurrence-free. The performance of two previously developed prognostic models of circulating miRNAs [16,17] was shown to be comparable among different histological types of NSCLC; thus, it is of particular interest to evaluate the predictive potential of the present miRNA panel for NSCLC. In addition, it is critical to integrate different types of biomarkers, such as ctDNAs, proteins, autoantibodies [11,12,13], or additional circulating prognostic miRNAs [36,37], with the present miRNA panel by comparing their performance side by side in the same sample sets, which will allow for the development of an optimal biomarker model that can be used to more accurately assess the aggressiveness of LUAD.

Our study has some limitations. First, we conducted a retrospective case–control study using a limited number of samples. Therefore, potential confounding factors, such as subject demographics, the presence of comorbidities, sample collection procedure, and storage time, were not fully matched. The lack of validated endogenous miRNA controls hampers the assessment of sample quality, which may have been affected by these confounding factors [15,38]. Nevertheless, the three-miRNA panel was indeed validated in an independent test set where samples were collected and stored under different protocols for different durations, suggesting the biological relevance of the miRNA panel developed in this study. Second, because our study focused on predicting outcome in patients with early-stage LUAD, the performance of the three-miRNA panel should also be evaluated in patients with advanced LUAD. Third, plasma samples were collected from only two institutions in Japan and the United States. Therefore, the generalizability of the results should be evaluated in future prospective and multi-institutional cohort studies, including LUAD patients with ethnicities beyond Asian and Caucasian. In addition, to implement the panel in clinical practice, an optimal cutoff value of the panel should be determined and validated in a prospective and clinical setting to assess the predictive accuracy of the panel. Fourth, the molecular mechanisms underlying the elevated levels of plasma miR-23a-3p, miR-320c, and miR-125b-5p remain largely unknown. These include oncogenic functions in LUAD cells, mechanisms of their release into the extracellular space and circulation, and potential regulation of biological functions of recipient cells in a paracrine or endocrine manner. Elucidating the biological role of these miRNAs in LUAD recurrence will identify novel therapeutic targets.

## 4. Materials and Methods

### 4.1. Human Plasma Samples

Whole blood was drawn from all subjects in EDTA vacuum tubes. Plasma was immediately isolated via serial centrifugations at 1200× *g* at room temperature for 12 min and then at 1200× *g* at 4 °C for 12 min. The plasma was stored at −80 °C until use. For initial discovery studies using a miRNA microarray, a pool of plasma samples was constituted from three stage I LUAD cases with recurrence (rec group: females, average age of 56 years, range of 48–65 years) and three stage I LUAD cases without recurrence (non-rec group: females, average age of 63 years, range of 48–75 years). These plasma samples were collected at MD Anderson Cancer Center prior to any treatment. Patients in the rec group had recurrence within two years after curative surgery, while those in the non-rec group were not diagnosed with recurrence during the follow-up period of six years. A pool of plasma samples from twenty female healthy controls [39] was used as the control group. Plasma samples obtained between February 2010 and October 2012 from 85 stage I LUAD patients with (*n* = 16) and without (*n* = 69) recurrence from Nagoya University were used for the initial validation of individual miRNAs and model development (validation set) (Table 2). An independent set of plasma samples, collected between June 2003 and February 2012 at MD Anderson Cancer Center from 57 patients with stage I and II LUAD with (*n* = 24) and without (*n* = 33) recurrence, was used for testing the resulting microRNA panel (test set) (Table 2). Disease stage was determined based on the American Joint Committee on Cancer’s tumor–node–metastasis staging (AJCC 7th Edition).

### 4.2. Plasma RNA Isolation

Total RNA was isolated from 200 μL of plasma using a Total RNA Purification Kit (Norgen, Thorold, ON, Canada), according to the manufacturer’s protocol. Prior to RNA extraction, 10 fmole of cel-miR-54 (Thermo Fisher, Carlsbad, CA, USA) was added to each sample as a spike-in control. Total RNA was eluted in RNase-free water, quantified using a NanoDrop 2000 spectrophotometer (Thermo Scientific, Carlsbad, CA, USA), and stored at −80 °C until use.

### 4.3. microRNA Microarray

miRNA profiling was performed using an Affymetrix microRNA 4.0 Array (Santa Clara, CA, USA) consisting of 2578 human miRNAs annotated in miRBase, v2.0. Briefly, 200 ng of total RNA was labeled with biotin using the FlashTag™ Biotin HSR RNA Labeling Kit (Affymetrix, Sunnyvale, CA, USA) and then hybridized overnight with the array, according to the manufacturer’s protocol. After washing and staining, the array was scanned with a GeneChip Scanner 3000 7G (Affymetrix), with the intensity of fluorescence calculated using the GeneChip Command Console Software package, v4.0 (Affymetrix). The raw intensity values were background-corrected, log_2_-transformed, and quantile-normalized using robust multichip analysis (RMA). Raw data of publicly available Affymetrix microRNA v4.0 Array datasets of H1299 LUAD cell lines (GSM1904747, GSM1904748, GSM1904749) [20] and peripheral blood mononuclear cells (PBMCs) from a healthy control (GSM1923351) [21] were processed as well using RMA.

### 4.4. Quantitative Real-Time PCR Analysis of LUAD Cell Lines

The human LUAD cell lines NCI-H3255, NCI-HCC2935, HCC827, DFCI032, NCI-H2228, DFCI024, NCI-H2030, HCC4019, NCI-H1395, and NCI-H838 were cultured in RPMI 1640 medium (GE Healthcare Life Sciences, Saint Louis, MO, USA) containing 10% fetal bovine serum (FBS) (Gemini, West Sacramento, CA, USA). The cell lines were authenticated by short tandem repeat (STR) analysis at the MD Anderson Characterized Cell Line Core facility (Houston, TX, USA) and confirmed to be mycoplasma-free with the use of a MycoAlert™ PLUS Mycoplasma Detection Kit (cat. #LT07-710, Lonza, Morristown, NJ, USA). All LUAD cell lines were provided by Dr. Adi Gazdar. Total RNA was isolated using an RNeasy Mini Kit (cat. #74102, Qiagen, Germantown, MD, USA), following the manufacturer’s instructions. cDNA was prepared using a High-Capacity cDNA Reverse Transcription Kit (Applied Biosystems, Waltham, MA, USA) or TaqMan Advanced miRNA cDNA Synthesis Kit (Applied Biosystems). A TaqMan qPCR assay was performed with the use of an Applied Biosystems™ QuantStudio™ 6 Flex Real-Time PCR System and the TaqMan Universal Master Mix II, no UNG (Applied Biosystems), or TaqMan Fast Advance master mix (Applied Biosystems), with FAM™-labeled TaqMan probes for miR-320c, miR-4532, miR-6511a-3p, miR-4745-5p, miR-4459, miR-3935, and RNU44 (Life Technologies, Frederick, MD, USA). Each sample was run in triplicate. Ct values for each miRNA were calculated and normalized to the Ct value for RNU44 (ΔCt).

### 4.5. Quantitative Real-Time PCR Analysis of Plasma Samples

Expression of plasma miRNAs in the validation and test sets was evaluated using a TaqMan MicroRNA assay kit, according to the manufacturer’s instructions. cDNA was prepared using target miRNA-specific reverse transcription (RT) primers (Life Technologies) and a High-Capacity cDNA Reverse Transcription Kit (Applied Biosystems). A TaqMan qPCR assay was performed with an Applied Biosystems™ QuantStudio™ 6 Flex Real-Time PCR System using the TaqMan Universal Master Mix II, no UNG (Applied Biosystems), with FAM™-labeled TaqMan probes for miR-23a-3p, miR-23b-3p, miR-191-5p, miR-185-5p, miR-151a-3p, miR-320c, miR-21-5p, miR-125b-5p, miR-30d-5p, miR-197-3p, and cel-miR-54 (Life Technologies). Each sample was run in triplicate. Ct values for each miRNA were calculated and normalized to the Ct value for cel-miR-54 (ΔCt).

### 4.6. CEA ELISA

Plasma levels of CEA were measured using a Luminex kit (HCCBP1MAG-58K, HSCRMAG-32K, Millipore, Saint Charles, MO, USA), according to the manufacturer’s instructions. Each sample was assayed in duplicate, and absorbance was determined with a calibrated Bio-Plex machine (Bio-Plex MAGPIX System, Bio-Rad, Hercules, CA, USA).

### 4.7. Development of miRNA Panel

All possible combinations of ten miRNAs were explored to select the best logistic regression model to discriminate between LUAD patients with and without recurrence based on the Akaike information criterion (AIC). A total of 1022 logistic regression models were fitted, and the combination of miR-320c and miR-125b-5p yielded the smallest AIC. In addition, a model combining miR-23a-3p and miR-125b-5p was developed with forward selection. Therefore, a three-marker miRNA panel containing miR-23a-3p, miR-320c, and miR-125b-5p was used as the final model. miRNA panel scores for each patient were generated based on the following justification: 13.344 + 0.30409 × log_2_ (miR-23-a-3p expression level) + 0.7029 × log_2_ (miR-320c expression level) + 0.99357 × log_2_ (miR-125b-5p expression level). To visualize recurrence and survival probability over time, a hazard constrained natural spline (HCNS) approach based on the aforementioned Cox models was used in combination with monotone smoothing splines [40,41].

### 4.8. Statistical Analyses

Categorical data were compared using Fisher’s exact test and continuous variables with a two-sided unpaired *t*-test. A one-sided Mann–Whitney U test was used to determine *p* values for comparisons of plasma miRNA levels between LUAD patients with and without recurrence, based on the alternative hypothesis that the miRNAs tested in this study were increased in plasma from patients with recurrence compared to those without. Survival analysis was performed with the validation and test sets using Kaplan–Meier survival curves. A log-rank test was used to evaluate the statistical significance of differences between survival curves. Disease-free survival and overall survival were defined as the time from the date of the initial surgery to the date of recurrence or death and last follow-up, respectively, at which point the data were censored. For overall survival, only death from disease was considered as an incidence factor. The proportionality of hazard assumption of a Cox regression was assessed for both recurrence (*p* = 0.331) and survival (*p* = 0.597) using Grambsch and Therneau’s method. Statistical analysis was conducted using the Prism 9 package (GraphPad). A statistical significance level of 0.05 was used for all statistical analyses.

## 5. Conclusions

In conclusion, we identified and validated circulating miR-23a-3p and miR-320c as novel biomarkers for predicting recurrence in patients with early-stage LUAD. Furthermore, a circulating miRNA panel comprising miR-23a-3p, miR-320c, and miR-125b-5p was also demonstrated to be an independent predictor of DFS and OS in early-stage LUAD. These findings provide rationale for further investigation to stratify early-stage LUAD patients using blood-based biomarkers to increase the ability to provide more personalized care.

## Figures and Tables

**Table 1:** miRNAs increased in pretreatment plasma of stage I LUAD patients with recurrence.

miRNA	Ratio of Rec vs. Non Rec	Ratio of Rec vs. Healthy Control	Ratio of H1299 vs. PBMC	Average in 34 LUAD Cell Lines (Log_2_ Intensity)	Average Ct Values in 10 LUAD Cell Lines
miR-23a-3p	3.48	5.86	2.11	5.60	-
miR-23b-3p	1.67	3.36	1.88	5.31	-
miR-191-5p	1.60	5.26	1.87	1.72	-
miR-185-5p	1.76	15.90	1.58	1.01	-
miR-151a-3p	2.13	2.70	3.45	0.98	-
miR-130b-3p	1.70	2.05	3.16	2.06	-
miR-23a-5p	1.98	1.55	1.51	−5.81	-
miR-361-5p	3.63	4.23	1.89	−6.01	-
miR-320c	2.56	1.62	1.60	NA	26.9
miR-4532	2.84	51.08	1.61	NA	>35
miR-6511a-3p	1.83	1.88	1.55	NA	>35
miR-4745-5p	2.39	3.41	1.93	NA	Not detected
miR-4459	8.29	12.61	1.80	NA	Not detected
miR-3935	2.44	1.73	2.12	NA	Not detected
miR-574-5p	14.70	37.16	1.59	NA	Not available

**Table 2:** Clinical characteristics of plasma sample sets.

Characteristics	Total	Recurrence	Non-Recurrence	*p* Value
		No. (%)	No. (%)	
Validation set				
Total	85	16 (18.8)	69 (81.2)	
Gender				
Male	43	8 (18.6)	35 (81.4)	>0.9999
Female	42	8 (19.0)	34 (81.0)	
Age				
Range, years (mean ± SD)		49–77 (68.9 ± 7.9)	47–84 (68.5 ± 6.7)	0.8258
>65 years	63	13 (20.6)	50 (79.4)	0.5458
<65 years	22	3 (13.6)	19 (86.4)	
Smoking status				
Smoker (current, former)	42	7 (16.7)	35 (83.3)	0.7825
Non-smoker	43	9 (20.9)	34 (79.1)	
CEA				
Mean ± SD, ng/mL		2.78 ± 3.88	2.75 ± 3.68	0.9753
Test set				
Total	57	24 (42.1)	33 (57.9)	
Gender				
Male	25	11 (44.0)	14 (56.0)	>0.9999
Female	32	13 (40.6)	19 (59.4)	
Age				
Range, years (mean ± SD)		32–79 (64.5 ± 10.5)	50–87 (66.9 ± 9.9)	0.3910
>65 years	31	15 (48.4)	16 (51.6)	0.4197
<65 years	26	9 (34.6)	17 (65.4)	
Smoking status				
Smoker (current, former)	47	22 (46.8)	25 (53.2)	0.1661
Non-smoker	10	2 (20.0)	8 (80.0)	
Stage (AJCC 7th Edition)				
I	36	10 (27.8)	26 (72.2)	0.0058
II	21	14 (66.7)	7 (33.3)	
CEA				
Mean ± SD, ng/mL		1.93 ± 2.03	1.40 ± 2.01	0.3313

## Data Availability

The raw data that support the findings of this study are available from the corresponding author upon reasonable request.

## Supplementary Materials

The following supporting information can be downloaded at https://www.mdpi.com/article/10.3390/ijms25042331/s1.

## Abbreviations

LUAD: lung adenocarcinoma; miRNA, microRNA; AUC, area under the curve; NSCLC, non-small cell lung cancer; AJCC, the American Joint Committee on Cancer; ctDNA, circulating tumor DNA; EDTA, ethylenediaminetetraacetic acid; PBMC, peripheral blood mononuclear cell; FBS, fetal bovine serum; STR, short tandem repeat; CEA, carcinoembryonic antigen; AIC, Akaike information criterion; HCNS, hazard constrained natural spline; qRT-PCR, quantitative real-time PCR; CI, confidence interval; OS, overall survival; DFS, disease-free survival; PGC-1α, Peroxisome proliferator-activated receptor gamma coactivator 1-alpha; G6PC, Glucose-6-phosphatase catalytic subunit 1; MTSS1, Metastasis suppressor protein 1; RKIP, Raf Kinase Inhibitory Protein; EpCAM, Epithelial cell adhesion molecule; EV, extracellular vesicle; EGFR, Epidermal growth factor receptor; ALK, ALK receptor tyrosine kinase; PD-1, Programmed cell death 1; TGF-β, transforming growth factor β; CD, cluster of differentiation; BLIMP-1, B-Lymphocyte-Induced Maturation Protein 1.
