# Supplementary material for: Circulating microRNA Panel for Prediction of Recurrence and Survival in Early-Stage Lung Adenocarcinoma
Source: Int J Mol Sci. 2024 Feb 16;25(4):2331. doi: 10.3390/ijms25042331 (PMC10888571; doi:10.3390/ijms25042331)
Supplement: Supplementary file 1 [file ijms-25-02331-s001.zip › ijms-2781810-supplementary.pdf]

Supplementary Table S1. Adjuvant chemotherapy and recurrence in test set

Adjuvant chemotherapy*	Total	Recurrence no. (%)	Non-recurrence no. (%)	P value†
**Stage I**				
Yes	2	1 (50.0)	1 (50.0)	0.4773
No	31	8 (25.8)	23 (74.2)	
**Stage II**				
Yes	13	10 (76.9)	3 (23.1)	0.1736
No	7	3 (42.9)	4 (57.1)	

*Information was not available for three stage I and one stage II LUAD patients.
†P values were calculated using Fisher's exact test.
